# Job Autonomy and Innovation in Healthcare and Human Services: Pathways Through Appraisal, Engagement, and Burnout

**DOI:** 10.3390/healthcare14040437

**Published:** 2026-02-09

**Authors:** Luke Pederson, Julie M. Slowiak

**Affiliations:** Department of Psychology, University of Minnesota Duluth, Duluth, MN 55812, USA; pede0604@d.umn.edu

**Keywords:** job autonomy, innovative work behavior, healthcare and human service professionals, cognitive appraisal, work engagement, job burnout, job demands–resources model, organizational climate, resilience, leadership

## Abstract

**Highlights:**

**What are the main findings?**

- Job autonomy significantly predicted innovative work behavior among healthcare and human service professionals, with mediation through cognitive appraisal, work engagement, and job burnout.
- Burnout exerted a negative effect on innovative work behavior, indicating that high autonomy alone cannot offset the detrimental impact of strain.

**What are the implications of the main findings?**

- Leaders in healthcare and human service organizations can foster innovation by enhancing autonomy while shaping appraisal processes to maximize engagement and minimize burnout.
- Structuring jobs and climates to support autonomy provides a pathway for organizations to strengthen workforce resilience and innovative capacity in response to social and organizational challenges.

**Abstract:**

**Background/Objectives**: Healthcare and human service organizations face mounting pressures to adapt to social and public health challenges while maintaining quality care. Innovative work behavior among healthcare and human service professionals is critical to organizational resilience. Prior research suggests that job autonomy fosters innovative work behavior, but the mechanisms underlying this relationship remain unclear. This study examined how cognitive appraisal, work engagement, and job burnout mediate the relationship between job autonomy and innovative work behavior. **Methods**: A non-experimental, cross-sectional online survey was conducted with 607 healthcare and human service professionals in the United States. Validated measures assessed job autonomy, cognitive appraisal, work engagement, job burnout, and innovative work behavior. Serial mediation analyses were performed using Hayes’ PROCESS macro (Model 6) with bootstrapping (*n* = 5000). Work innovation was included as a covariate to control for organizational climate effects. **Results**: Job autonomy was positively related to innovative work behavior, work engagement, and both challenge and hindrance appraisal. The direct relationship between job autonomy and job burnout was mixed, significant in the hindrance appraisal model but not in the challenge appraisal model. Mediation analyses revealed that challenge and hindrance appraisal significantly influenced the pathways from job autonomy to work engagement and job burnout, which in turn mediated the job autonomy—innovative work behavior relationship. Burnout had a significant negative effect on innovative work behavior, whereas engagement strengthened the positive relationship between job autonomy and innovative work behavior. The full model explained 65.12–67.73% of the variance in innovative work behavior. **Conclusions**: Job autonomy is a critical driver of innovative work behavior among healthcare and human service professionals, operating through appraisal, engagement, and burnout. Building on previous research, this study extends prior evidence by clarifying when autonomy enables professionals to thrive and innovate, and when it risks contributing to burnout. Findings underscore the importance of appraisal-based interventions and autonomy-supportive climates to sustain workforce well-being and organizational innovation.

## 1. Introduction

Healthcare and human service organizations, including hospitals, clinics, and residential facilities, face mounting pressures such as population growth, globalization of knowledge, and emerging public health threats. To remain viable, they must continually adapt and develop new services [1]. At the same time, healthcare and human service professionals are expected to improve the quality of care they provide, often with limited personal and job resources to manage excessive demands. To meet these challenges, healthcare and human service professionals frequently engage in innovative work behavior—actions beyond formal job requirements, such as collaborating, exchanging knowledge, and generating solutions to organizational and social issues (e.g., domestic violence, substance abuse, infectious diseases, access to care) [2].

Healthcare and human service settings are also characterized by high emotional demands and emotional labor, as professionals routinely manage client interactions and complex interpersonal situations [3]. In addition, healthcare and human service roles involve intense service delivery pressures and heavy workloads that can strain available resources [4]. These pressures are compounded by strict professional and regulatory constraints that influence how practitioners make decisions and carry out their work [5,6]. At the same time, organizations in these sectors face continual pressure to innovate in order to improve service quality and respond to evolving community needs [7]. Such conditions heighten the importance of job resources like autonomy, which enable professionals to exercise judgment, adapt to rapidly changing circumstances, and generate innovative solutions to never-ending service challenges. Understanding how autonomy functions in these demanding care environments is therefore essential for identifying conditions that support or hinder innovative work behavior.

Engaging in innovative work behavior benefits both organizations and professionals. For healthcare and human service organizations, innovative work behavior enhances productivity, innovation, and profitability. For healthcare and human service professionals, it fosters well-being by providing a sense of purpose and motivation to improve performance [8]. Given these outcomes, researchers have examined how job autonomy influences innovative work behavior, as healthcare and human service professionals often make critical, life-changing decisions about patient care [9]. Recent studies suggest that cognitive appraisal (i.e., how healthcare and human service professionals perceive autonomy) and work engagement may explain the strength and direction of this relationship [9,10]. For example, healthcare and human service professionals report higher work engagement when they have greater autonomy, motivating them to engage in innovative work behavior despite heavy workloads [11,12,13]. Importantly, the positive relationship between job autonomy and work engagement is stronger when healthcare and human service professionals appraise autonomy-related demands as opportunities for growth rather than hindrances to workflow. Together, these findings suggest a central process: job autonomy shapes how professionals appraise their work, which in turn influences their engagement, burnout, and ultimately their innovative work behavior.

Although prior research shows job autonomy is positively related to innovative work behavior, with cognitive appraisal and work engagement functioning as mediators [9,14,15], less is known about how appraisal explains conditions under which job autonomy facilitates or inhibits innovative work behavior by influencing both work engagement and job burnout. The Job Characteristics Model (JCM) identifies autonomy as a core job resource that enhances intrinsic motivation and creativity [16], while the Job Demands–Resources (JD–R) model positions autonomy as a resource that can motivate employees or, under certain conditions, contribute to strain [17]. These frameworks suggest that autonomy may not uniformly benefit healthcare and human service professionals; instead, its effects may depend on how autonomy-related demands are interpreted. This gap is especially salient in emotionally demanding service environments, where the same autonomy that enables flexibility may also introduce responsibility, ambiguity, or strain.

Cognitive appraisal theory provides a useful lens for understanding this variability. Challenge appraisals frame autonomy as an opportunity for growth, whereas hindrance appraisals frame it as an obstacle [18,19]. Because autonomy can simultaneously introduce discretion and responsibility, healthcare and human service professionals may appraise it as both challenging and hindering [9]. These appraisals may shape whether autonomy leads to engagement and innovation or contributes to burnout and withdrawal. Thus, examining appraisal processes may clarify why autonomy sometimes enables professionals to thrive and at other times imposes strain.

In addition, emerging research suggests that autonomy may have non-linear effects, where very high levels of autonomy can introduce ambiguity, decision burden, or role overload [20,21]. Although the present study focuses on linear relationships for theoretical parsimony and consistency with prior JD–R research, acknowledging this possibility highlights the importance of understanding how employees interpret autonomy-related demands.

Accordingly, the aim of this study is to clarify how job autonomy influences innovative work behavior among healthcare and human service professionals by examining the roles of cognitive appraisal, work engagement, and job burnout as serial mediators. This study addresses a key gap by integrating appraisal theory with the JCM and the JD–R framework to explain when and why autonomy supports or undermines innovation in high-demand service settings. In doing so, the study contributes theoretically by identifying appraisal-based mechanisms linking job autonomy to both motivational and strain pathways and contributes practically by offering insight into how leaders can design roles that promote innovation without sacrificing well-being.

## 2. Literature Review

### 2.1. Innovative Work Behavior and Job Autonomy

Innovative work behavior involves recognizing problems, generating ideas, and collaborating with coworkers and stakeholders to implement solutions [22]. Researchers conceptualize innovative work behavior as a dynamic, multi-stage process that includes idea generation, idea promotion, and idea realization [22]. These stages require employees to notice opportunities, mobilize support, and translate ideas into practice, activities that depend heavily on job resources, discretion, and supportive work conditions. In healthcare and human service settings, innovative work behavior is particularly critical because professionals must adapt to evolving client needs, regulatory requirements, and complex service environments. Prior research shows that innovative work behavior in these sectors enhances organizational learning, service quality, and responsiveness to community needs [23,24,25].

Because innovative work behavior requires both creativity and proactive problem solving, researchers often examine job characteristics to understand conditions that foster it. According to the JCM [16], autonomy is a core job resource that enhances intrinsic motivation by increasing experienced responsibility for outcomes. Autonomy allows employees to determine how to perform tasks, explore new ideas, and tailor solutions to organizational needs [23]. Through the psychological states outlined in the JCM (i.e., experienced meaningfulness, responsibility, and knowledge of results), autonomy supports creativity, experimentation, and initiative, all of which are foundational to innovative work behavior. Thus, because autonomy enhances intrinsic motivation and provides discretion to adapt work processes, it is expected to promote innovative work behavior.

The relationship between job autonomy and innovative work behavior is complex and shaped by employee and organizational factors [9,20]. Job autonomy refers to the extent to which employees control how they execute tasks and manage competing demands [26,27]. High autonomy can encourage creativity and innovative work behavior by reframing demands as opportunities [13,28]. Yet autonomy may also impose additional burdens, particularly in unpredictable, high-stakes contexts [12,29]. For example, child-care workers may benefit more from autonomy than elderly care workers, who face morally challenging situations. Thus, professionals may perceive autonomy as either a resource that supports performance or a demand that hinders workflow.

Recent studies further suggest that autonomy may exhibit non-linear or even adverse effects under certain conditions. Excessive autonomy can reduce coordination, enable free riding, or foster unethical shortcuts, ultimately hindering innovative work behavior [20,21,30,31]. These findings indicate that autonomy may be most beneficial within an optimal range, where discretion supports creativity without eroding accountability or shared responsibility. Because autonomy generally enhances intrinsic motivation and provides flexibility to pursue new ideas, we hypothesized that:

**H1.**  *Job autonomy is positively related to innovative work behavior.*

### 2.2. Job Autonomy, Work Engagement, and Burnout

Within the JD-R model [17], work engagement occurs when employees possess and invest sufficient resources to meet demands. Adequate resources foster active coping, job satisfaction, and organizational commitment, which encourage extra-role behaviors like innovative work behavior [32,33]. Autonomy functions as a motivational resource because it supports self-determination, competence, and meaningfulness, all of which are key drivers of engagement. Research typically finds a positive relationship between job autonomy and work engagement, moderated by resource availability [34,35]. Because autonomy provides control, supports self-regulation, and enables employees to align work with their strengths, the JD–R model predicts that autonomy should increase work engagement. As such, we hypothesized that:

**H2.**  *Job autonomy is positively related to work engagement.*

Job burnout, in contrast, emerges when employees face excessive demands with insufficient resources [17]. Burnout diverts resources away from task accomplishment, reducing capacity for innovative work behavior [14,36]. Research generally shows an inverse relationship between job autonomy and job burnout; greater autonomy helps employees manage demands and experience fewer burnout symptoms [21,26]. However, autonomy can also increase planning demands, reducing empowerment in unpredictable contexts [20]. This duality underscores ongoing debate about whether autonomy primarily empowers or overwhelms healthcare and human service professionals, highlighting the need for further investigation. Because autonomy can reduce strain by increasing control over work processes, JD–R predicts that autonomy should be negatively related to burnout. Therefore, we hypothesized that:

**H3.**  *Job autonomy is negatively related to job burnout.*

### 2.3. Cognitive Appraisal as a Pathway to Well-Being and Innovation

Cognitive appraisal refers to employees’ interpretation of work stressors as either beneficial or detrimental [18]. Challenge appraisals frame autonomy as an opportunity for growth, learning, and mastery, while hindrance appraisals frame it as an obstacle that interferes with goal attainment [19]. Job autonomy can be appraised simultaneously as both a challenge and hindrance, reflecting its dual potential for resource gain or depletion [9]. In healthcare and human service settings, where decisions carry moral weight, emotional demands are high, and work is interdependent, autonomy may be especially ambiguous. Because appraisal influences whether autonomy is experienced as energizing or overwhelming, it is a critical mechanism linking autonomy to well-being and innovation. Given that job autonomy can lead to two different appraisals, we hypothesized that:

**H4a.**  *Job autonomy is positively related to challenge appraisal.*

**H4b.**  *Job autonomy is positively related to hindrance appraisal.*

Research shows job autonomy’s effects on work engagement and job burnout depend on appraisal [10,33]. Challenge appraisal fosters enthusiasm and mitigates demands, increasing work engagement, while hindrance appraisal reduces confidence and energy [9,37]. Less research examines appraisal’s role in the job autonomy–job burnout relationship, but evidence suggests challenge appraisal weakens the link between demands and burnout, whereas hindrance appraisal strengthens it [21]. Because appraisal determines whether autonomy is interpreted as a resource or a demand, it is expected to mediate autonomy’s effects on engagement and burnout. Thus, we hypothesized that:

**H5a.**  *Challenge appraisal mediates the relationship between job autonomy and work engagement.*

**H5b.**  *Hindrance appraisal mediates the relationship between job autonomy and work engagement.*

**H6a.**  *Challenge appraisal mediates the relationship between job autonomy and job burnout.*

**H6b.**  *Hindrance appraisal mediates the relationship between job autonomy and job burnout.*

Appraisals of job autonomy also shape energy and engagement, influencing innovative work behavior. Challenge appraisal fosters work engagement, which promotes innovative work behavior, while hindrance appraisal reduces work engagement and limits innovative work behavior [9,38]. Job burnout may also mediate this relationship. Challenge appraisal reduces burnout, freeing resources for innovative work behavior, whereas hindrance appraisal increases exhaustion, depleting resources and inhibiting creativity [39,40]. Because appraisal influences both well-being and motivation, it is expected to channel autonomy into either thriving or depletion, influencing whether professionals innovate or withdraw. Therefore, we hypothesized that:

**H7a.**  *Challenge appraisal and work engagement serially mediate the relationship between job autonomy and innovative work behavior.*

**H7b.**  *Hindrance appraisal and work engagement serially mediate the relationship between job autonomy and innovative work behavior.*

**H8a.**  *Challenge appraisal and job burnout serially mediate the relationship between job autonomy and innovative work behavior.*

**H8b.**  *Hindrance appraisal and job burnout serially mediate the relationship between job autonomy and innovative work behavior.*

### 2.4. Present Study

**Figure 1:**
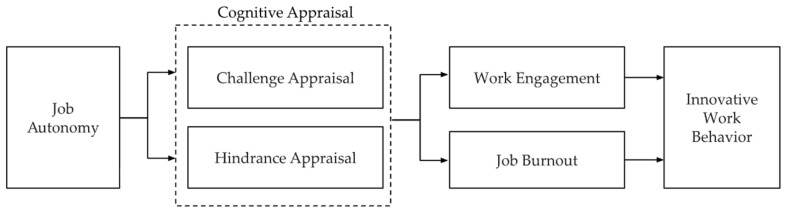
Hypothesized Conceptual Model. The model illustrates the proposed relationships among job autonomy, cognitive appraisal (challenge appraisal and hindrance appraisal), work engagement, job burnout, and innovative work behavior. Direct and mediated pathways are mapped to the study’s hypotheses. This figure depicts parallel serial mediation pathways in which job autonomy influences innovative work behavior through both positive (challenge appraisal, higher work engagement, lower job burnout) and negative (hindrance appraisal, lower work engagement, higher job burnout) appraisal processes.

This study extended prior work by examining how healthcare and human service professionals’ cognitive appraisals of job autonomy influence well-being outcomes (engagement and burnout) and, in turn, innovative work behavior. Building on Baig et al. [9], we tested a serial mediation model with a broader professional demographic, incorporating both challenge and hindrance appraisal pathways. By integrating the JCM, the JD–R framework, and cognitive appraisal theory, this study clarifies when autonomy enables professionals to thrive and innovate, and when it risks contributing to burnout and withdrawal. The hypothesized conceptual model is illustrated in Figure 1, which maps job autonomy to cognitive appraisal, to work engagement/job burnout, and ultimately to innovative work behavior via parallel serial mediation paths.

## 3. Materials and Methods

### 3.1. Study Sample

Participants were 607 healthcare and human service professionals aged 18–73 years (*M* = 33.80, *SD* = 7.24) who were employed in the United States in roles where they actively provided care or services to patients or clients. Most identified as Caucasian/White (*n* = 453, 74.60%), male (*n* = 318, 52.40%), and worked in physicians’ offices (*n* = 84, 13.80%). More than half of the participants worked directly with patients or clients (*n* = 388, 63.90%) and held a managerial or supervisory position within their organization (*n* = 372, 61.30%). Participants were generally well-educated, with most holding at least a bachelor’s degree, and had an average of 6–10 years of experience in their field. A full breakdown of sociodemographic and job-related characteristics is provided in Appendix A.

### 3.2. Study Design and Procedure

This study employed a non-experimental, cross-sectional online survey design. Data were collected between June and late August 2024, following approval from the University of Minnesota’s Institutional Review Board. Participants were recruited through email lists affiliated with professional societies, messages forwarded by colleagues within their professional networks (e.g., local, state, or regional membership organizations), and contacts working in their organizations.

Recruitment messages included a brief description of the study and information about inclusion criteria, eligibility requirements, time commitment, and the participation incentive. They also contained a link to the web-based survey hosted on Qualtrics and a request to forward the message and survey link to others who met eligibility requirements. Participants were informed that participation was voluntary, responses would remain confidential, results would be reported in an aggregate and anonymous form, and they could withdraw at any time without penalty. After providing electronic consent, participants completed screening items to confirm eligibility, followed by questions assessing sociodemographic and job-related characteristics, as well as measures of job autonomy, cognitive appraisal, work engagement, job burnout, and innovative work behavior.

The study posed minimal risk to participants and adhered to the ethical guidelines of the American Psychological Association. Participants experiencing high levels of job burnout may have felt slight emotional discomfort when responding to burnout-related items. To mitigate this, a statement at the end of the survey encouraged participants to contact their employee health or assistance program if they needed support. A link to the Mayo Clinic’s [41] article, *Job Burnout: How to Spot It and Take Action*, was also provided to help participants understand the antecedents and consequences of burnout. Finally, participants could provide an email address, via a link unassociated with their survey responses, to enter a prize drawing for one of 25 $20 Amazon eGift Cards.

### 3.3. Measures

#### 3.3.1. Sociodemographic and Job-Related Characteristics

The survey included 11 items assessing sociodemographic and job-related characteristics. These items covered age, gender, race, ethnicity, education level, industry, certification/license, years of experience with the current employer, clinical role status, and managerial/supervisory status. Responses provided information on participants’ personal and job-related characteristics. In addition, relationships between these characteristics and the primary study variables were examined to determine whether any should be included as covariates in the analyses.

#### 3.3.2. Job Autonomy

Job autonomy was assessed using three items from Pee and Chau [42], adapted from Morris and Venkatesh’s [27] version of the Job Diagnostic Survey [16]. Sample items included: “To what extent does your job have autonomy?” and “To what extent does your job give you the opportunity for independence and freedom in how you do the work?” Participants rated each item on a seven-point Likert-type scale ranging from 1 (*not at all*) to 7 (*to a very great extent*), with 4 representing a moderate level. A total score was calculated by averaging item scores; higher scores indicated greater autonomy. Internal consistency for this sample was *α* = 0.80.

#### 3.3.3. Cognitive Appraisal

Cognitive appraisal was assessed using a six-item scale developed by LePine et al. [18]. Three items measured challenge appraisal and three measured hindrance appraisal. Sample items included: “Working to fulfill the demands of my job helps to improve my personal growth and well-being” (challenge appraisal) and “Working to fulfill the demands of my job thwarts my personal growth and well-being” (hindrance appraisal). Participants rated each item on a five-point Likert-type scale ranging from 1 (*strongly disagree*) to 5 (*strongly agree*). Subscale scores were calculated by averaging item scores; higher scores indicated higher levels of challenge or hindrance appraisal. Internal consistency values were *α* = 0.74 for challenge appraisal and *α* = 0.67 for hindrance appraisal.

#### 3.3.4. Work Engagement

Work engagement was assessed using the three-item version of the Utrecht Work Engagement Scale (UWES-3 [43]). Each item measured one dimension of work engagement: vigor, dedication, or absorption. Sample items included: “At my work, I feel bursting with energy” (vigor) and “I am enthusiastic about my job” (dedication). Participants rated each item on a seven-point Likert-type scale ranging from 0 (*never*) to 6 (*always*). A total score was calculated by averaging item scores; higher scores indicated greater work engagement. Internal consistency for this sample was *α* = 0.83.

#### 3.3.5. Job Burnout

Job burnout was assessed using the 12-item Burnout Assessment Tool (BAT-12 [44]), which conceptualizes burnout across four dimensions: exhaustion, mental distance, cognitive impairment, and emotional impairment. Sample items included: “At work, I feel mentally exhausted” (exhaustion) and “I struggle to find any enthusiasm for my work” (mental distance). Participants rated each item on a five-point Likert-type scale ranging from 1 (*never*) to 5 (*always*). An overall burnout score was calculated by averaging item scores; higher scores indicated greater prevalence of burnout symptoms. Internal consistency for this sample was *α* = 0.92.

#### 3.3.6. Innovative Work Behavior

Innovative work behavior was assessed using Janssen’s [22] nine-item scale that includes items related to idea generation, idea promotion, and idea realization. Sample items included: “I often generate original solutions for problems” (idea generation) and “I mobilize support for innovative ideas” (idea promotion). Participants rated the frequency of engaging in these behaviors on a seven-point Likert-type scale ranging from 1 (*never*) to 7 (*always*). An overall innovative work behavior score was calculated by averaging item scores; higher scores indicated greater innovative work behavior. Internal consistency for this sample was *α* = 0.92.

#### 3.3.7. Work Innovation

Work innovation was assessed using a seven-item scale from Wipulanusat et al. [45] that includes items related to individual creativity and team innovation. Sample items included: “I am able to explore new ideas about how I do my job” (individual creativity) and “People in my work group analyze their work to look for ways of doing a better job” (team innovation). Participants rated each item on a five-point Likert-type scale ranging from 1 (*strongly disagree*) to 5 (*strongly agree*). An overall work innovation score was calculated by averaging item scores; higher scores indicated greater work innovation. Internal consistency for this sample was *α* = 0.82. Work innovation was assessed to control for potential effects of organizational climate on innovative work behavior.

#### 3.3.8. Commitment Request and Attention Checks

To improve data quality, participants completed one commitment request item, one textual attention check, and one instructional attention check. The commitment request item stated: “We care about the quality of our survey data. For us to get the most accurate measures of your responses, it is important that you provide thoughtful answers to each question in this survey. Do you commit to providing thoughtful answers to the questions in this survey?” Response options were: “I can’t promise either way,” “Yes, I will,” or “No, I won’t.” Only participants who selected “Yes, I will” were able to proceed; others were directed to the end of the survey and removed from the dataset.

The textual attention check was an open-ended item instructing participants to type the word “orange” in a box. The instructional attention check asked participants to select “Agree” from a five-point Likert-type scale ranging from *Strongly Disagree* to *Strongly Agree*. Only those who responded correctly were able to proceed; others were directed to the end of the survey and removed.

### 3.4. Statistical Analysis

Data were cleaned and analyzed using SPSS (version 29.0.1.0). Internal consistency of study measures was assessed using Cronbach’s alpha coefficients. Pearson product–moment correlations were computed to examine associations among variables, test the relational hypotheses, and identify potential covariates. A Monte Carlo simulation using Schoemann et al.’s [46] web application (*shinyapp*) was conducted to estimate the required minimum sample size for a model with two serial mediators. Using a standard alpha level (*α* = 0.05) and desired power of 0.80, with medium effects assumed for paths a1, b2, and d and small effects assumed for paths a2, b1, and c′, the recommended minimum sample size was 134 participants.

Construct validity was evaluated using confirmatory factor analysis (CFA) with maximum likelihood estimation, specifying seven latent factors: job autonomy, challenge appraisal, hindrance appraisal, work engagement, job burnout, innovative work behavior, and work innovation. Model fit was assessed using *χ*^2^, CFI, TLI, RMSEA, and SRMR, and standardized factor loadings were examined to evaluate convergent validity. Potential common method variance was assessed using Harman’s single-factor test and by comparing the hypothesized measurement model with a single-factor CFA model. Hypotheses were tested using serial mediation analyses conducted with Hayes’ PROCESS macro (Model 6, version 4.2 [47]), employing 5000 bootstrap samples to generate bias-corrected 95% confidence intervals. Heteroscedasticity-robust standard errors were used, and variables were mean-centered prior to analysis [38]. Given the cross-sectional design, mediation results are interpreted as evidence of indirect associations consistent with theory rather than causal effects [47,48].

## 4. Results

### 4.1. Data Cleaning and Response Quality Assessment

De-identified survey responses were downloaded from Qualtrics, and a response quality assessment was conducted prior to analysis. Qualtrics’ ExpertReview response quality feature [49] was used to evaluate 985 responses. A total of 372 responses were removed because they were identified as fraudulent (e.g., spam, bots, duplicates), failed to agree to participate, were ineligible (e.g., failed screening items, reported impossible age, missing age data), failed or did not complete attention checks, did not finish the survey, or provided nonsensical responses.

Outliers were detected during regression assumption testing using SPSS casewise diagnostics. Six additional participants were removed. The final analytic sample included 607 participants.

### 4.2. Preliminary Analyses

Table 1 presents the means, standard deviations, correlations, and Cronbach’s alpha values for the primary study variables. The average job autonomy score (*M* = 4.72, *SD* = 1.24) indicated moderate autonomy. Average challenge (*M* = 3.88, *SD* = 0.76) and hindrance (*M* = 3.31, *SD* = 0.91) appraisal scores reflected moderate tendencies. The average work engagement score (*M* = 4.01, *SD* = 1.32) also reflected moderate engagement. The overall innovative work behavior score was *M* = 4.79, *SD* = 1.17. The average work innovation score was *M* = 3.80, *SD* = 0.66. This moderate work innovation score may account for variance in innovative work behavior not explained by the primary study variables.

Schaufeli et al. [50] established clinically validated cut-off scores for severe burnout based on European samples. Scores < 2.54 indicated no burnout, scores > 2.96 indicated burnout symptoms, and scores between 2.54 and 2.96 indicated risk. Using these cutoffs, most participants reported no burnout (*n* = 360, 59.30%), followed by severe burnout symptoms (*n* = 163, 26.90%) and risk for burnout (*n* = 84, 13.80%).

The average job burnout score was *M* = 2.35, *SD* = 0.80. Because U.S. norms for the BAT-12 were not yet available, burnout levels were defined relative to ±1 SD of the mean: ≤1.55 = low, 1.56–3.15 = moderate, and ≥3.16 = high. Most participants reported moderate burnout (*n* = 388, 63.90%), followed by high (*n* = 118, 19.40%) and low (*n* = 101, 16.60%).

Using Cohen’s [51] guidelines, the Pearson’s *r* correlation analysis revealed significant correlations among study variables: a large, positive relationship between job autonomy and innovative work behavior (supports H1); a large, positive relationship between job autonomy and work engagement (supports H2); a small, negative relationship between job autonomy and job burnout (supports H3); a large, positive relationship between job autonomy and challenge appraisal (supports H4a); and a medium, positive relationship between job autonomy and hindrance appraisal (supports H4b).

### 4.3. Measurement Model and Common Method Bias Checks

The seven-factor CFA demonstrated acceptable fit: *χ*^2^(719) = 1817.38, *p* < 0.001; CFI = 0.92; TLI = 0.91; RMSEA = 0.05, 90% CI [0.047, 0.053]; SRMR = 0.04. All standardized factor loadings were statistically significant (*p* < 0.001) and were generally moderate to strong, with most exceeding 0.60 and many above 0.70, supporting convergent validity. One hindrance appraisal item loaded somewhat lower (0.38) but was retained for theoretical reasons. In addition, Harman’s single-factor test indicated that the first factor accounted for 34.8% of the total variance, below the conventional 50% threshold. Finally, the seven-factor CFA measurement model demonstrated significantly better fit than a single-factor model (Δ*χ*^2^ = 3232.4, Δdf = 21, *p* < 0.001). Together, these results provide confidence in the distinctiveness of the study constructs and support the validity of subsequent mediation analyses.

### 4.4. Mediation Analyses

#### 4.4.1. Challenge Appraisal Model

**Figure 2:**
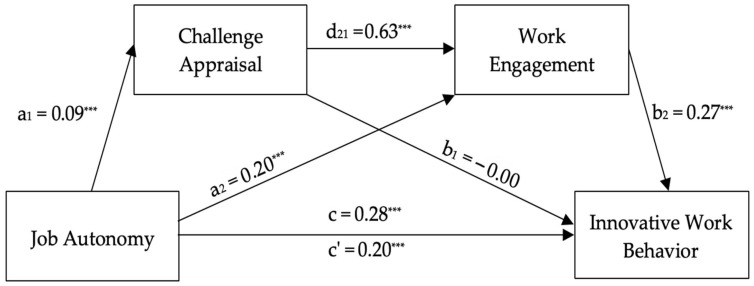
Unstandardized regression coefficients for the relationship between job autonomy and innovative work behavior as mediated by challenge appraisal and work engagement. Note: *N* = 607. *** *p* < 0.001. Work innovation was included as a covariate; its path is not displayed for simplicity.

Figure 2 shows the serial mediation model with challenge appraisal and work engagement. Job autonomy significantly predicted innovative work behavior (*c* = 0.2757, *t* = 7.81, *p* < 0.001, CI = 0.2064–0.3451), work engagement (*a*_2_ = 0.2047, *t* = 5.16, *p* < 0.001, CI = 0.1267–0.2826), and challenge appraisal (*a*_1_ = 0.0927, *t* = 4.28, *p* < 0.001, CI = 0.0501–0.1352), supporting H1, H2, and H4a, respectively. The indirect effect of job autonomy on work engagement through challenge appraisal was significant (*a*_1_*d*_21_ = 0.0161, Boot CI = 0.0078–0.0266), supporting H5a. The full model explained 66.81% of innovative work behavior variance after controlling for work innovation, *R*^2^ = 0.67, *F*(4, 602) = 426.75, *p* < 0.001, supporting H7a. The direct effect of job autonomy on innovative work behavior remained significant (*c*’ = 0.2037, *t* = 6.01, *p* < 0.001, CI = 0.1372–0.2703), indicating partial mediation.

**Figure 3:**
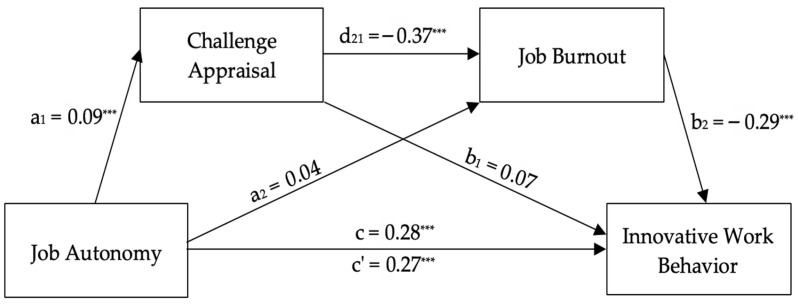
Unstandardized regression coefficients for the relationship between job autonomy and innovative work behavior as mediated by challenge appraisal and job burnout. Note: *N* = 607. *** *p* < 0.001. Work innovation was included as a covariate; its path is not displayed for simplicity.

Figure 3 shows the model with challenge appraisal and job burnout. Job autonomy significantly predicted innovative work behavior (*c* = 0.2757, *t* = 7.81, *p* < 0.001, CI = 0.2064–0.3451) and challenge appraisal (*a*_1_ = 0.0927, *t* = 4.28, *p* < 0.001, CI = 0.0501–0.1352), supporting H1 and H4a, respectively. The direct effect of job autonomy on job burnout was not significant (*a*_2_ = 0.0383, *t* = 1.32, *p* = 0.186, CI = −0.0185–0.0951), failing to support H3, despite a small, negative correlation (*r* = −0.25, *p* < 0.001). The indirect effect of job autonomy on job burnout through challenge appraisal was significant (*a*_1_*d*_21_ = 0.0097, Boot CI = 0.0046–0.0165), supporting H6a. The full model explained 65.12% of innovative work behavior variance after controlling for work innovation, *R*^2^ = 0.65, *F*(4, 602) = 337.00, *p* < 0.001, supporting H8a. The direct effect of job autonomy on innovative work behavior remained significant (*c*’ = 0.2709, *t* = 8.19, *p* < 0.001, CI = 0.2059–0.3359), indicating partial mediation.

#### 4.4.2. Hindrance Appraisal Model

**Figure 4:**
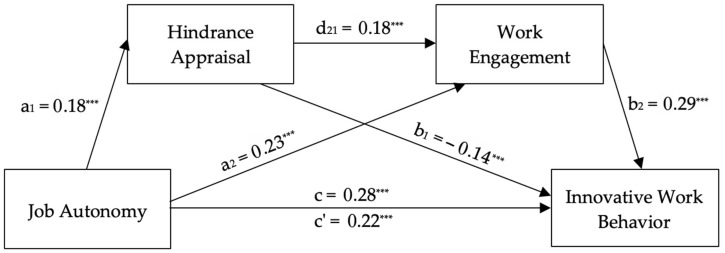
Unstandardized regression coefficients for the relationship between job autonomy and innovative work behavior as mediated by hindrance appraisal and work engagement. Note: *N* = 607. *** *p* < 0.001. Work innovation was included as a covariate; its path is not displayed for simplicity.

Figure 4 shows the model with hindrance and work engagement. Job autonomy significantly predicted innovative work behavior (*c* = 0.2757, *t* = 7.81, *p* < 0.001, CI = 0.2064–0.3451), work engagement (*a*_2_ = 0.2314, *t* = 5.57, *p* < 0.001, CI = 0.1529–0.3135), and hindrance appraisal (*a*_1_ = 0.1777, *t* = 4.76, *p* < 0.001, CI = 0.1040–0.2496), supporting H1, H2, and H4b, respectively. The indirect effect of job autonomy on work engagement through hindrance appraisal was significant (*a*_1_*d*_21_ = 0.0093, CI = 0.0040–0.0161), supporting H5b. The full model explained 67.73% of innovative work behavior variance after controlling for work innovation, *R*^2^ = 0.68, *F*(4, 602) = 402.34, *p* < 0.001, supporting H7b. The direct effect of job autonomy on innovative work behavior remained significant (*c*’ = 0.2228, *t* = 7.00, *p* < 0.001, CI = 0.1603–0.2853), indicating partial mediation.

**Figure 5:**
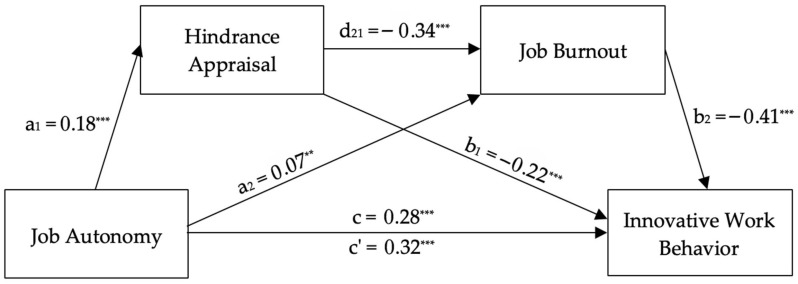
Unstandardized regression coefficients for the relationship between job autonomy and innovative work behavior as mediated by hindrance appraisal and job burnout. Note: *N* = 607. ** *p* < 0.05. *** *p* < 0.001. Work innovation was included as a covariate; its path is not displayed for simplicity.

Figure 5 shows the model with hindrance appraisal and job burnout. Job autonomy significantly predicted innovative work behavior (*c* = 0.2757, *t* = 7.81, *p* < 0.001, CI = 0.2064–0.3451), job burnout (*a*_2_ = 0.0653, *t* = 2.18, *p* < 0.05, CI = 0.0065–0.1242), and hindrance appraisal (*a*_1_ = 0.1777, *t* = 4.76, *p* < 0.001, CI = 0.1044–0.2509), supporting H1, H3, and H4b, respectively. The indirect effect of job autonomy on job burnout through hindrance appraisal was significant (*a*_1_*d*_21_ = 0.0251, Boot CI = 0.0139–0.0381), supporting H6b. The full model explained 67.18% of innovative work behavior variance after controlling for work innovation, *R*^2^ = 0.67, *F*(4, 602) = 337.45, *p* < 0.001, supporting H8b. The direct effect of job autonomy on innovative work behavior remained significant (*c*’ = 0.3173, *t* = 10.63, *p* < 0.001, CI = 0.2587–0.3760), indicating partial mediation.

## 5. Discussion

The purpose of this study was to examine how cognitive appraisal (challenge and hindrance), work engagement, and job burnout mediate the relationship between job autonomy and innovative work behavior among healthcare and human service professionals. By focusing on these mechanisms, we sought to clarify how autonomy contributes to thriving and innovation in high-demand care settings. Overall, the findings confirmed that job autonomy consistently predicted innovative work behavior, with partial mediation through appraisal, work engagement, and job burnout. Notably, the direct relationship between job autonomy and job burnout was mixed, providing partial support for H3. The hypothesized indirect effects emerged in the hindrance appraisal model but not consistently in the challenge appraisal model, suggesting that autonomy’s protective effect against burnout depends on how demands are appraised.

The positive association between job autonomy and innovative work behavior is consistent with Baig et al. [9], whose work provided the foundation for the present study. Their findings demonstrated that autonomy enhances employees’ capacity to manage demands and conserve resources, thereby facilitating innovation. Our results extend this evidence to healthcare and human service professionals, showing that autonomy similarly enables them to generate, promote, and implement ideas. This conclusion is further supported by prior studies linking autonomy to innovative outcomes across diverse contexts [21,34,39,52]. The JCM [16] also identifies autonomy as a critical job resource that fosters creativity and collaboration, a perspective echoed by Asurakkody and Shin [23].

Job autonomy’s positive relationship with work engagement aligns with the JD–R model [17] and with research showing that autonomy enhances vigor and dedication [43]. The mixed support for the relationship between job autonomy and job burnout reflects nuanced findings in the literature. While job autonomy is often described as a buffer against job burnout [26], its effect may be contingent on appraisal processes [10]. Gerich and Weber [53] found that challenge appraisal is more strongly linked to satisfaction, which may explain why job autonomy’s direct effect on job burnout was nonsignificant in the challenge appraisal model. Although autonomy is generally viewed as a resource, some research also suggests that its effects may be non-linear [20,21], with very high levels potentially creating ambiguity or strain; the present study did not test such curvilinear patterns. Future research should examine whether diminishing-returns effects emerge in autonomy’s relationship with burnout and innovation.

Notably, the positive association between hindrance appraisal and work engagement was unexpected, given theoretical models that typically associate hindrance appraisals with reduced motivation and poorer outcomes [10,39,54]. One possible explanation is that in high-demand healthcare and human service settings, even demands appraised as hindering may still require sustained effort and involvement [26,37,53], thereby maintaining engagement despite elevated strain. This pattern suggests that engagement and burnout may coexist under certain conditions [37,40,55], highlighting the importance of examining both motivational and strain pathways simultaneously.

Beyond these contributions, several methodological strengths enhance confidence in the present findings. Including work innovation as a covariate allowed us to account for organizational climate influences on innovative work behavior, improving model fit and isolating the unique contribution of job autonomy. This approach reflects prior work showing that organizational climate and structure can motivate or discourage employees from engaging in innovation [45,56]. The large and diverse sample of healthcare and human service professionals further strengthens the generalizability of the results across varied organizational contexts. Data quality was also supported through the use of a commitment item and attention checks, which reduced careless responding.

Finally, most measures demonstrated strong internal consistency, with only the hindrance appraisal subscale showing a slightly lower alpha (α = 0.67). Although below the conventional 0.70 threshold, this value is not unusual for very short scales. Cronbach’s alpha is highly sensitive to scale length, and brief three-item measures routinely yield lower estimates even when items are psychometrically sound [57,58,59]. Taken together, these methodological strengths reduce measurement error, support the robustness of the findings, and provide a strong foundation for interpreting the study’s theoretical and practical implications.

### 5.1. Theoretical Implications

Building on the preceding findings, this study contributes to theory by clarifying how job autonomy operates through cognitive appraisal and well-being processes to influence innovative work behavior in high-demand care settings. Across both models, the persistence of significant direct effects alongside significant indirect effects indicates that job autonomy influences innovative work behavior both directly and indirectly through appraisal, engagement, and burnout. This pattern is consistent with theoretical expectations from the JD-R model and appraisal theory, which posit that job resources can simultaneously exert direct motivational effects and indirect effects through cognitive and affective pathways. The presence of partial mediation suggests that autonomy has unique motivational value beyond its indirect effects through well-being pathways. Because the study used cross-sectional data, these mediation results should be interpreted as indirect associations rather than causal processes.

The mediating roles of challenge and hindrance appraisal are consistent with the Challenge–Hindrance Stressor Framework [54] and with studies showing that appraisal shapes whether demands foster growth or hinder well-being [10,18]. Challenge appraisal appears to channel job autonomy into thriving, increasing engagement and reducing job burnout, whereas hindrance appraisal may weaken job autonomy’s protective effects against strain while still promoting work engagement. Together, these findings highlight appraisal as a key mechanism linking job autonomy to both thriving and strain in healthcare and human service workforces. Additionally, job burnout may account for discrepancies in the strength of the job autonomy—innovative work behavior relationship that cannot be explained by work engagement.

Although alternative model structures, such as treating work engagement, job burnout, and innovative work behavior as parallel outcomes, are theoretically plausible, our serial mediation approach aligns with appraisal theory and the JD–R framework, which conceptualize engagement and burnout as proximal motivational and strain-related mechanisms through which job resources influence innovative behavior. This structure allows us to examine how appraisal and well-being processes jointly channel the effects of autonomy into innovative behavior in healthcare and human service contexts. That said, future research should directly compare serial and parallel outcome models using longitudinal designs to test temporal ordering.

### 5.2. Practical Implications

The findings reveal how job autonomy can empower healthcare and human service professionals to accomplish their tasks while conserving resources needed to engage in innovative work behavior. Because healthcare and human service organizations must adapt to markets shaped by social issues, employees are required to remain flexible in their work approaches [1,60]. One strategy is job crafting, which involves increasing resources and challenging demands while reducing hindering demands [61,62]. This approach aligns with the JD-R model [17] by helping employees acquire resources to improve performance and minimize cognitive and emotional demands that contribute to job burnout [62]. Job crafting also allows employees to reshape their work to better match their strengths and support professional development, thereby enhancing effectiveness within organizational structures and creating more opportunities for innovation.

The results further suggest that job autonomy encourages job crafting by providing freedom to redesign jobs in response to environmental changes. Within the JD-R framework, job crafting functions as a resource that can increase satisfaction and work engagement by enabling employees to create challenging yet manageable demands [55,62]. Job crafting also helps employees adjust to unpredictable environments, and autonomy itself may expand as professionals engage in innovative work behavior [60]. However, leaders must adopt approaches that emphasize employee well-being and development to ensure that autonomy and job crafting are directed toward organizational goals rather than solely personal interests [63].

The findings provide leaders with a clearer understanding of how job characteristics such as job autonomy influence innovative work behavior through appraisal and well-being (work engagement and job burnout). Challenge appraisal appears to strengthen the motivational benefits of autonomy, whereas hindrance appraisal may increase burnout and reduce the resources available for innovation. This understanding can guide leaders in redesigning jobs so autonomy is perceived as less threatening. Encouraging employees to coordinate on projects and change management efforts can foster communication and trust, which are essential for collaboration [64,65,66]. Such collaboration can promote equitable task distribution, reduce workload strain, and free resources for innovation [64]. These efforts also foster perceived task and outcome interdependence, improving adaptive team functioning and strengthening collective innovation [65].

Finally, leaders can reshape organizational climates to mitigate demands associated with job autonomy, such as role ambiguity or work interruptions, thereby maximizing challenge appraisal and work engagement while minimizing hindrance appraisal and job burnout [10,12]. When job autonomy is supported in this way, employees are more likely to recognize its benefits for themselves and their organizations [67]. Overall, this study provides a foundation for leaders to enhance the innovative capacity of healthcare and human service organizations by structuring jobs and shaping climates that encourage autonomy without compromising well-being.

### 5.3. Limitations and Future Research

Several limitations of this study should be acknowledged, and they point directly to opportunities for future research. First, the non-experimental, cross-sectional design prevents causal inference. Therefore, the mediation results should be interpreted as evidence of theoretically consistent indirect associations rather than causal processes [47,48]. Although the proposed ordering is grounded in appraisal theory and the JD–R model, alternative causal directions and reciprocal relationships cannot be ruled out. Longitudinal or cross-lagged designs would allow researchers to examine how these relationships evolve over time and in response to organizational changes [28,68].

Second, the use of convenience sampling limits representativeness and generalizability. Our sample may not fully reflect the broader population of healthcare and human service professionals, and confounding variables may have influenced the results. Future studies should employ more systematic recruitment strategies to ensure diverse representation across roles and organizational contexts, which may provide insight on how different environmental factors (e.g., incentives, pay systems/structures, organizational values) impact variables in our model.

Third, our measure of cognitive appraisal assessed participants’ general appraisal of job stressors rather than appraisal of autonomy-specific demands. This prevented us from examining whether appraisal of job autonomy itself led to variations in work engagement and job burnout. Developing autonomy-specific appraisal measures would improve construct validity. For example, Gerich and Weber [53] measured challenge and hindrance appraisals of specific demands such as time pressure and task complexity. Adapting such approaches to autonomy-related demands could provide more precise insights into how appraisal shapes outcomes. Qualitative methods, such as semi-structured interviews or phenomenological analyses, may also help explain why professionals appraise job autonomy differently and identify contextual factors shaping appraisal [3,69].

Fourth, reliance on self-report measures may have introduced bias, including social desirability and recall issues. Behavioral measures could complement self-reports in future studies. For example, innovative work behavior might be assessed by the number of ideas generated and implemented, work engagement by observed task persistence, and job burnout by behavioral indicators such as withdrawal or off-task behavior [70]. Incorporating such measures would reduce bias and provide more objective indicators of the constructs studied.

Finally, cultural differences in burnout thresholds complicate interpretation. Schaufeli et al. [50] established European cutoffs for burnout severity, but de Beer et al. [71] found that Japanese residents reported higher BAT-12 scores than European samples, highlighting the need for cross-cultural validation. Future research should examine whether U.S. professionals experience burnout differently and establish culturally appropriate norms.

In addition to addressing these limitations, future studies should explore leadership styles and organizational climate as moderators of the autonomy–innovation relationship. Transformational and servant leadership, for example, may strengthen the positive effects of autonomy, while transactional leadership may constrain them [3,60,63]. Research on leadership methods such as motivational interviewing could also reveal how leaders empower professionals to reframe autonomy as a challenge rather than a hindrance, thereby increasing engagement and reducing burnout. Cross-cultural and multi-level studies would further clarify how organizational climate and leadership interact with autonomy to shape innovation.

## 6. Conclusions

In conclusion, this study demonstrates that job autonomy is a critical driver of innovative work behavior among healthcare and human service professionals, operating through appraisal, work engagement, and job burnout. Autonomy consistently predicted innovative work behavior, and the mediation analyses showed that challenge and hindrance appraisals, work engagement, and job burnout each contributed to explaining this relationship. The presence of significant direct and indirect effects indicates that autonomy exerts motivational influence beyond its effects on appraisal and well-being, highlighting its unique role in supporting innovation.

Notably, autonomy’s protective effect against burnout was conditional. Challenge appraisal reduced burnout and strengthened engagement, whereas hindrance appraisal increased burnout and weakened the resources available for innovation. These findings underscore the importance of appraisal processes in shaping outcomes. Burnout exerted a significant negative effect on innovation, indicating that high autonomy alone cannot fully offset the detrimental impact of strain.

Building directly on Baig et al. [9], our findings extend prior evidence by showing how appraisal pathways help explain when autonomy enables professionals to thrive and innovate and when it risks contributing to burnout and withdrawal in demanding care environments. By clarifying these mechanisms in a large, diverse sample and controlling for work innovation, our study advances theory and offers actionable guidance for designing autonomy-supportive roles and climates that sustain engagement while mitigating burnout. These contributions position job autonomy not only as a lever for innovation but as a construct whose effects depend on appraisal, reinforcing the need for appraisal-informed strategies to strengthen workforce resilience.

## Figures and Tables

**Table 1:** Means, Standard Deviations, Intercorrelations, and Cronbach’s Alpha Values for Study Variables.

Variable	*M*	*SD*	1	2	3	4	5	6	7
1. Job autonomy	4.72	1.24	**0.80**						
2. Challenge appraisal	3.88	0.76	0.46 **	**0.74**					
3. Hindrance appraisal	3.31	0.91	0.34 **	0.34 **	**0.67**				
4. Work engagement	4.01	1.32	0.52 **	0.65 **	0.35 **	**0.83**			
5. Job burnout	2.35	0.80	−0.25 **	−0.50 **	−0.48 **	−0.53 **	**0.92**		
6. Innovative work behavior	4.78	1.26	0.61 **	0.59 **	0.22 **	0.70 **	−0.51 **	**0.92**	
7. Work innovation	3.80	0.66	0.53 **	0.66 **	0.30 **	0.65 **	−0.47 **	0.75 **	**0.82**

Notes: *N* = 607. All correlation (Pearson’s *r*) values are based on listwise exclusion. ** *p* < 0.01 (2-tailed). Cronbach’s alpha values are shown in bold along the diagonal.

## Data Availability

The data presented in this study are available on request from the corresponding author due to the sharing of the dataset being restricted under the conditions of our Institutional Review Board (IRB) protocol, which prohibits public dissemination of participant-level data.

## Appendix A

**Table A1:** Sociodemographic and Job-Related Characteristics of Participants.

Characteristics and Categories	*n*	%
Age		
18–24	33	5.4
25–34	306	50.4
35–44	225	37.1
45–54	36	5.9
55–64	3	0.5
65+	4	0.7
Gender		
Male	318	52.4
Female	286	47.1
Transgender	2	0.3
Prefer not to answer	1	0.2
Race		
Alaskan Native	12	2.0
American Indian	30	4.9
Asian/Pacific Islander	2	0.3
African American/Black	80	13.2
Native Hawaiian	7	1.2
Other Pacific Islander	2	0.3
Caucasian/White	453	74.6
Prefer not to answer	3	0.5
Multiple races	18	3.0
Education		
Less than high school	7	1.2
High school or GED	11	1.8
Some college credit, but less than 1 year of college	35	5.8
1 or more years of college credit, no degree	49	8.1
Associates degree	73	12.0
Bachelor’s degree	161	26.5
Master’s degree	130	21.4
Professional degree beyond bachelor’s degree	78	12.9
Doctorate degree	51	8.4
Prefer not to answer	12	2.0
Industry		
Offices of physicians	84	13.8
Offices of dentists	71	11.7
Offices of chiropractors	41	6.8
Offices of optometrists	47	7.7
Offices of other health practitioners	45	7.4
Outpatient care centers	43	7.1
Home health care services	52	8.6
Other health care services	41	6.8
General medical and surgical hospitals, and specialty hospitals	30	4.9
Psychiatric and substance abuse hospitals	25	4.1
Nursing care facilities	36	5.9
Residential care facilities, without nursing	10	1.6
Individual and family services	26	4.3
Community food and housing, and emergency services	18	3.0
Vocational rehabilitation services	13	2.1
Child day care services	11	1.8
Other	14	2.3
Certification		
Yes	461	75.9
No	146	24.1
Years of Employment		
<1 year	10	1.6
1–2 years	75	12.4
3–5 years	253	41.7
6–10 years	210	34.6
11–15 years	34	5.6
16–20 years	6	1.0
>20 years	7	1.2
Missing	12	2.0
Direct Care		
No, not at all	8	1.3
Yes, less than half of work is direct care with patients/clients	207	34.1
Yes, majority of work is direct care with patients/clients	388	63.9
I’m not sure	3	0.5
Missing	1	0.2
Managerial Role		
Yes	372	61.3
No	233	38.4
Missing	2	0.3

Note: *N* = 607.
